# The Life Cycle and Life Span of Namibian Fairy Circles

**DOI:** 10.1371/journal.pone.0038056

**Published:** 2012-06-27

**Authors:** Walter R. Tschinkel

**Affiliations:** Department of Biological Science, Florida State University, Tallahassee, Florida, United States of America; University of Western Australia, Australia

## Abstract

In Namibia of southwestern Africa, the sparse grasslands that develop on deep sandy soils under rainfall between 50 and 100 mm per annum are punctuated by thousands of quasi-circular bare spots, usually surrounded by a ring of taller grass. The causes of these so-called “fairy circles” are unknown, although a number of hypotheses have been proposed. This paper provides a more complete description of the variation in size, density and attributes of fairy circles in a range of soil types and situations. Circles are not permanent; their vegetative and physical attributes allow them to be arranged into a life history sequence in which circles appear (birth), develop (mature) and become revegetated (die). Occasionally, they also enlarge. The appearance and disappearance of circles was confirmed from satellite images taken 4 years apart (2004, 2008). The frequency of births and deaths as a fraction of the total population of circles allowed the calculation of an approximate turnover rate, and from this, an estimate of circle lifespan. Lifespan appeared to vary with circle size, with small circles averaging about 24 years, and larger ones 43–75 years. Overall lifespan averaged about 41 yr. A second, independent estimate of lifespan was made by revisiting circles 2 to 9 years after their clear status had been confirmed. This resulted in a lifespan estimate of about 60 years. Any causal explanation of fairy circles must include their birth, development and death, their mean lifespan and the variation of their features under different conditions.

## Introduction

The eastern edge of the Namib Desert of southwestern Africa (the pro-Namib) is home to a mysterious phenomenon called “fairy circles”–nearly circular barren patches within a sparse matrix of small short-lived grass species (e.g. *Stipagrostis uniplumus* (Licht.) De Winter). The patches are often surrounded by a halo of taller grass (*Stipagrostis giessii Kers or S. hochstetteriana (Beck ex Hack.)*) and range from about 2 to 12 m in diameter. They are conspicuous in aerial or satellite photographs as well as on the ground, and occur by the hundreds of thousands in quasi-regular spacing [1] from southern Angola to northern South Africa wherever the soil is sandy and the rainfall is between 50 and 100 mm per annum [1], [2].

Several hypotheses of the causes of these circles were reviewed and evaluated by van Rooyen et al. [2] but to date, none are well supported and the formation of the circles remains a mystery. Although soil taken from the barren center did not inhibit germination of grass seed, it supported plant growth poorly, whereas that from the edge and matrix allowed growth [1]–[4], but these effects were not consistent for all areas or sampled years [2]. More recently, Jankowitz et al. [5] found that grass growth in pots was poor inside the circles but not the matrix when the pot was open below, but not when it was closed, and suggested a semi-volatile toxic factor. Neither macronutrient nor soil microbiota differences between circle and matrix soil could account for fairy circles [2], [6]. Other unsupported hypotheses included radioactivity, allelopathy and termite activity (all reviewed in [2]. Several authors [1], [3], [4], [7], [8], [9] proposed some version of causation by termites either through direct action, residual effect or emission of a toxic agent. However, Tschinkel [10] found no association between the nests or underground foraging tunnels of the endemic termite *Baucaliotermes hainseii* and fairy circles, nor have other termite species been found to be associated with fairy circles [2], [9].

Van Rooyen et al. [2] suggested that fairy circles result from environmental heterogeneity or self-organization or both. Self-organized vegetation patterns are widespread in arid lands and elsewhere, and Rietkerk et al. [11]–[12] as well as Couteron and Lejeune [13]–[15] proposed that such patterns are the result of nearby positive and distant negative feedbacks created by plants and physical processes occurring at different scales and intensities. Their models based on such feedbacks produced a wide range of patterns, ranging from spots to stripes to labyrinths and holes, corresponding to patterns seen in a range of real ecosystems. A non-linear mathematical analysis of arid zone vegetation interactions by Tlidi et al. [16] evolved patterns of localized bare spots whose distribution ranged from spatially independent, to self-organized, to randomly distributed, depending on the strength of interactions. Under some conditions, patterns similar to fairy circles were formed. Grasses grown in the laboratory under some conditions of water scarcity formed rings in agreement with feedback models [17].

Moll [4] and Albrecht et al. [1] described signs that fairy circles appear, evolve and “die.” Fewer than 5% of the circles in the area Albrecht et al. surveyed showed signs of either recent origin or revegetation, suggesting that fairy circles were a fairly long-lived phenomenon. Van Rooyen et al. [2] found several marked fairy circles 20 years later, and contested the suggestion of Moll [4] and Becker and Getzin [8] that fairy circles disappear after long droughts.

In the search for the causes of fairy circles, it seems important to know exactly what needs to be explained. The purpose of this paper is therefore to describe in greater detail the structure and range of variation of fairy circles in multiple habitats. In addition, the first estimates of the “life span” of fairy circles are presented, along with a detailed description of the stages of the life cycle. Any mechanism hypothesized as causing fairy circles must account for their size, spacing, appearance, longevity and disappearance, as well as the variation in these attributes in different habitats.

## Materials and Methods

### Study Area

**Figure 1:**
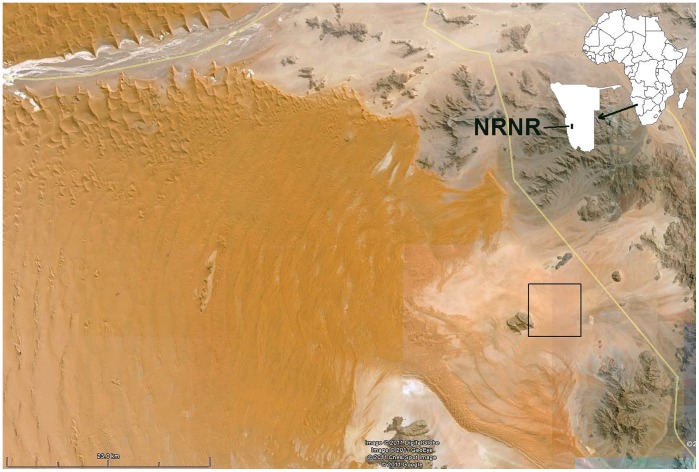
Google Earth image showing the location of the 2004 and 2008 satellite photographs used in this study. Inset shows the location of Namibia in Africa, and the Namib Rand Nature Reserve (NRNR) in Namibia.

The study area was located approximately 290 km SSW (azimuth of 203°) of Windhoek, Namibia in the Namib Rand Nature Reserve in the pro-Namib Desert at the eastern edge of the Namibian sand sea. The study was permitted under Ministry of Environment and Tourism permit No. 1422/2009. The study sites (Fig. 1) were all located between two rocky outcrops about 5 km apart, the Jagkop in the west and the Bushman Hills to the northeast. This area consisted of low dunes and sand plains that sloped gently to the east and was traversed by two vehicle tracks. Most of the subject fairy circles were located within 0.5 km of these tracks.

The climate of the Namib Rand Nature Reserve is extremely arid, with mild winters and hot summers (monthly mean temperatures: winter ∼11°C, summer ∼25°C). The mean annual rainfall for the Reserve is 70–80 mm, mostly as convective storms during the summer (December-May) that derive moisture from the Indian Ocean, and occasionally as a few mm during the winter. In 2011, a very wet year, rainfall totaled 335 mm as a result of unusually heavy rains during January to March. Almost no rain fell between May and January, as is typical.

### Satellite Photographs

**Figure 2:**
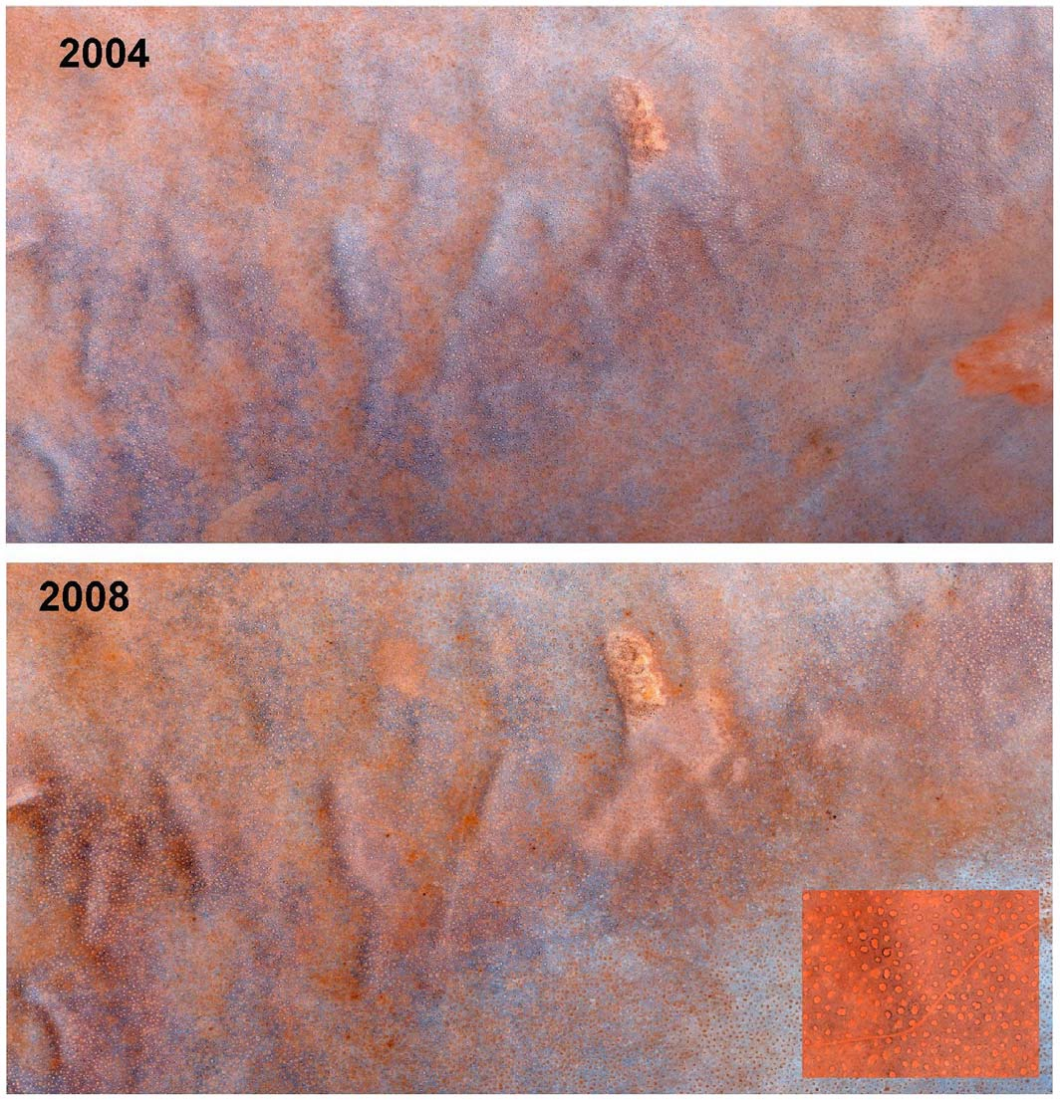
A section of the 2004 and 2008 satellite images covering much of the study area. Fairy circles are clearly visible. The inset in the lower right corner shows a section at a larger scale.

The basis of this study was a pair of georeferenced satellite visible spectrum, color photographs of a 5 by 5 km area, one taken in the morning of October 4, 2004 and the other, the morning of December 8, 2008. Both photographs had a resolution of 0.5 m, and were purchased from Digital Globe Imaging (ID: 052181792030_01 (2004) and 052181792040_01 (2008). The corner coordinates were: NW long. 15.929, lat. −24.953; NE long. 15.978, lat. −24.953; SE long. 15.978, lat. −24.998; SW long. 15.929, lat. −24.998. The photographs were added to GIS ArcMap 9.3 (ESRI) as layers. Thousands of fairy circles were visible (Fig. 2 shows about 20% of the entire image), and by toggling between the 2004 and 2008 layers, it was possible to search for differences between them. Because of variations in aspect and illumination, not all areas were amenable to easy interpretation, but in areas that were, there was many instances of changes.

When the image of a fairy circle was different in the two years, its coordinates were loaded into a Trimble Geoexplorer CE GPS instrument and the fairy circle or feature was visited on the ground and photographed. Similarly, long strolls through the landscape found many features of interest whose coordinates were collected on the GPS and by photograph so they could be located on the satellite photographs. In this way, a collection of ground-truthed features was accumulated, and these aided the interpretation of the satellite images.

These procedures resulted in the following categories of differences between the years: (1) circles absent in 2004 but appearing in 2008 (birth); (2) circles lacking a ring of taller grass in 2004, but with such a ring in 2008 (maturation); (3) circles that remained unchanged (stasis, the great majority of circles); (4) circles present in 2004 but distinctly larger in 2008 (growth); (5) circles with a ring, but whose center was distinctly less red than surrounding circles (dying); (6) circles detectable only by the ring of taller grass (ghosts), the area within the ring having been revegetated and indistinguishable from the matrix. All these phases are described in greater detail in the Results.

Variation of fairy circle structure, size and spacing was further elucidated with images covering the Namib Rand Nature Reserve and its environs downloaded from Google Earth. Image dates ranged from 2004 to 2011.

### Calculating the Life Span

The satellite photographs provided the data for estimating fairy circle life span in two ways. The initial method was targeted to “features of interest,” either noted on the satellite photos, or seen during peregrinations on the ground. Such features of interest included revegetated, enlarged or new circles, as deduced by a comparison of the feature in 2004 and 2008. All features were visited in 2009 and photographed. This provided a ground-truthing of the features seen on the images, and allowed the identification of features first seen on the ground with a feature in the photographs. Twenty eight sample areas (not to a constant scale) contained one or more ground-truthed features and were used to develop criteria for judging the life stage of all circles within each image, both in 2004 and 2008. Ground-truthing increased confidence in the judgment of life stage, and allowed better interpretation of image details. However, these images were not random samples of the study area as a whole, and could therefore be biased for the purpose of estimating circle life span.

In the second method, areas were selected from the satellite photos in a grid pattern, with each area at a scale of 1∶429 (176 m×90 m) and each containing about 50 to 80 circles. Each circle was assigned a number. A comparison of the image of the circle in 2004 and 2008 allowed determination of the life stage of the circle. Because this judgment was not always easy and clear, each value was also assigned a level of confidence from 1 (high) to 3. Life spans were estimated only from the first two levels of confidence. These gridded samples represented a reasonably random sample of the larger area in which the research was carried out.

In both cases, life span was calculated by assuming that the population of fairy circles was approximately in a steady state, and that births and deaths were therefore approximately equal. The life span was then the reciprocal of the fractional turnover rate, which was itself the average of the birth and death rate, divided by 4 because the images were taken 4 years apart. Because of the uncertainty inherent in assessing the states of circles, life span was also estimated from births and deaths alone (again, divided by 4), which sets each in turn to be equal to the other. Because each bookmark or image contained only a few births or deaths (or none), these were summed over all the samples for computation of the life span.

### Estimation of life Span from “Sold” Fairy Circles

**Figure 3:**
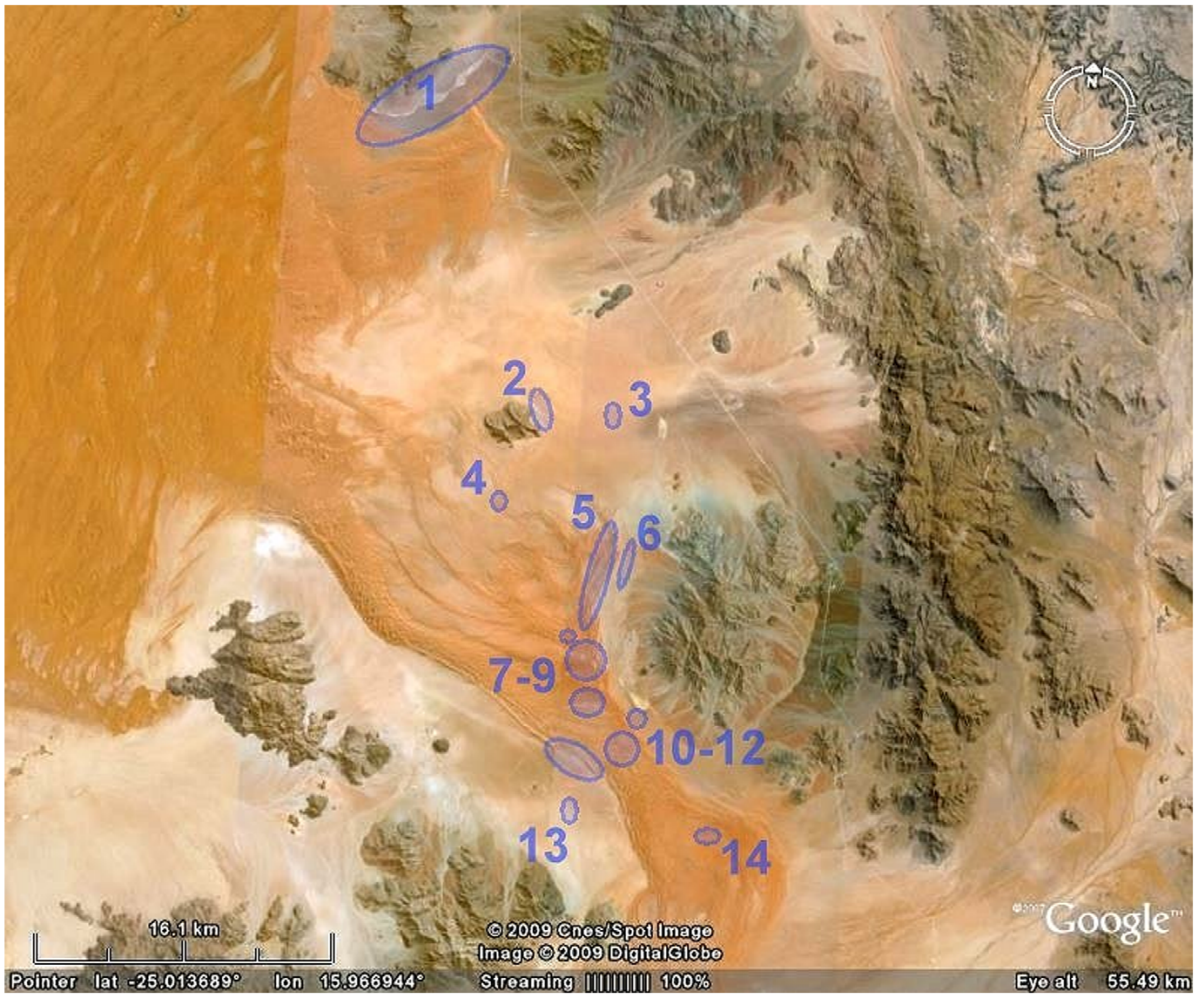
The Namib Rand region showing the locations of groups of “sold” fairy circles used to estimate circle lifespans.

The Namib Rand Nature Reserve raised money to support the conservation operations of the reserve by “selling” fairy circles to tourists visiting the Wolwedans ecotourism concession. The buyer, date of sale, and the GPS coordinates of each circle were recorded, and a ceramic numbered plaque was placed in the center of each sold circle. After the return of the author to the USA, we hired Michael and Ann Scott to find as many of these numbered, dated fairy circles as possible, and to record and photograph their current condition. Most of these fairy circles were “sold” in a limited number of areas, whose positions within the NRNR are shown in Fig. 3. Clusters occurred in several different landforms, including dry wash plain (cluster 1), mildly sloping sand plain (clusters 2, 3, 4), flat sand plain (clusters 6, 13), dunes, and dune slopes and interdune valleys (clusters 5, 7–12, 14). These data allowed the association of an elapsed time with each circle’s condition, and the calculation an approximate lifespan and turnover rate of circles (see below). The photographs also provided data on the type and height of vegetation around circle edges as well as in the surrounding matrix.

Frederick Huffer of the Department of Statistics, Florida State University provided an estimation of fairy circle lifespans from the “sold circle” data. The calculation used a standard parametric analysis of current status data. There were n  = 129 observations (fairy circles) for which we had complete data. For the i-th fairly circle (i  = 1; 2; : : :; 129) we know (t_i_; δ_i_) where t_i_ is the elapsed time in years between the date marked and the date revisited, and δ_i_ is the condition of the fairy circle at the time of revisiting with δ_i_  = 1 denoting “ghost” and δ_i_  = 0 denoting “clear”. We assume the fairy circles are independent with exponentially distributed lifetimes. Let β denote the mean lifespan (in years). The probability that a fairy circle will be clear (δ_i_  = 0) after an elapsed time of t_i_ years is e^-ti/β^, and the probability it will be a ghost is 1 - e^-ti/β^. The likelihood function for this data is obtained by taking the product of the individual probabilities of obtaining the observed values of δ_i_, i  = 1; : : :; 129, and is given by
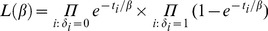
(1)The maximum likelihood estimate of β is the value β which maximizes L(β). An approximate 100(1 - α)% confidence interval for β is given by
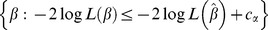
(2)where cα is the upper α quantile of a χ21 distribution, the chi-square distribution with one degree of freedom. This means that the confidence interval consists of all values β for which −2 log L(β) falls below the level −2 log L(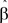) + cα. The upper and lower limits of the confidence interval are easily found (numerically) by solving for the two values of β at which the function −2 log L(β) crosses this level.

The expected fraction φ of fairy circles which will die (become ghosts) in any given one year period is φ  = 1– e^−1/β^ = _. The maximum likelihood estimate (MLE) of this fraction is.
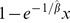
(3)where 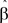 is the MLE of β given earlier. If (β_L_; β_U_) are the lower and upper limits of a confidence interval for β (at some given level), the corresponding confidence interval for the fraction φ is simply given by
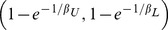
(4)A parametric proportional hazards (Cox-type) model analysis with area as a covariate was used to detect an effect, if any, of circle area on life span.

## Results

### The phases of the Life Cycle Of Fairy Circles–Birth, Maturation, Enlargement and Death

There was a great deal of variation in details of structure and condition of fairy circles, allowing circles to be categorized into a sequence from first appearance as a bare spot in the grassy matrix (birth), acquisition of a rim of tall bushman grass (with or without enlargement) (maturation), and revegetation of the bare area within the grass rim (death), and finally the disappearance of the grass circle, leaving an area indistinguishable from the matrix.

**Figure 4:**
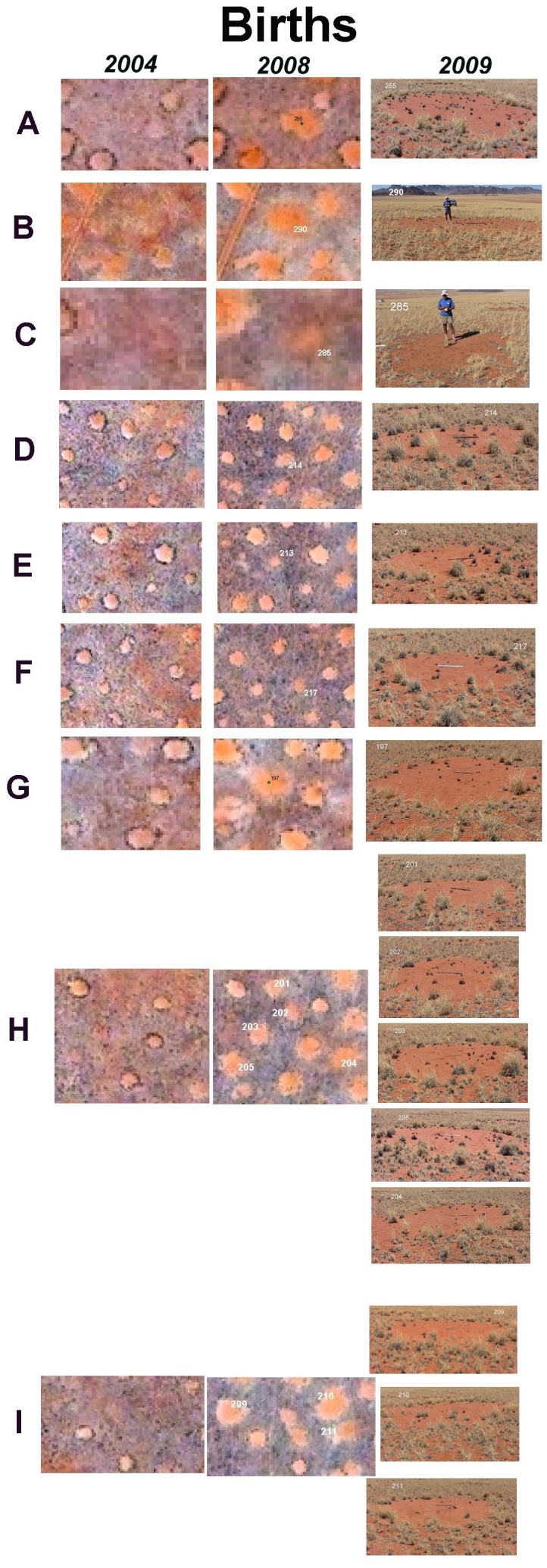
Satellite images from 2004 and 2008 showing the appearance (birth) of fairy circles, and their condition upon ground checking in 2009. Numbers in the 2008 images indicate circles not present in 2004, i.e. new circles. Panels H and I also each contain two unnumbered circles.

On the satellite images, newly formed circles were visible as a bright, reddish area in 2008, but this same spot was indistinguishable from the matrix in 2004 (Fig. 4A–I). When inspected on the ground in November 2009, these circles were distinguished by their flatness (old circles are concave [1]), the absence of a tall grass margin and the presence of dead clumps of grass within the bare area. In each satellite image extract, the tall bushman grass perimeter of existing circles is recognizable as a dark circle, which is absent on the new circles in 2008. However, a number of circles had acquired this tall perimeter by 2009, e.g. Fig. 4A, D, F and several in H and I). All of these new circles contained a few to many clumps of dead grass, mostly small bushman grass.

**Figure 5:**
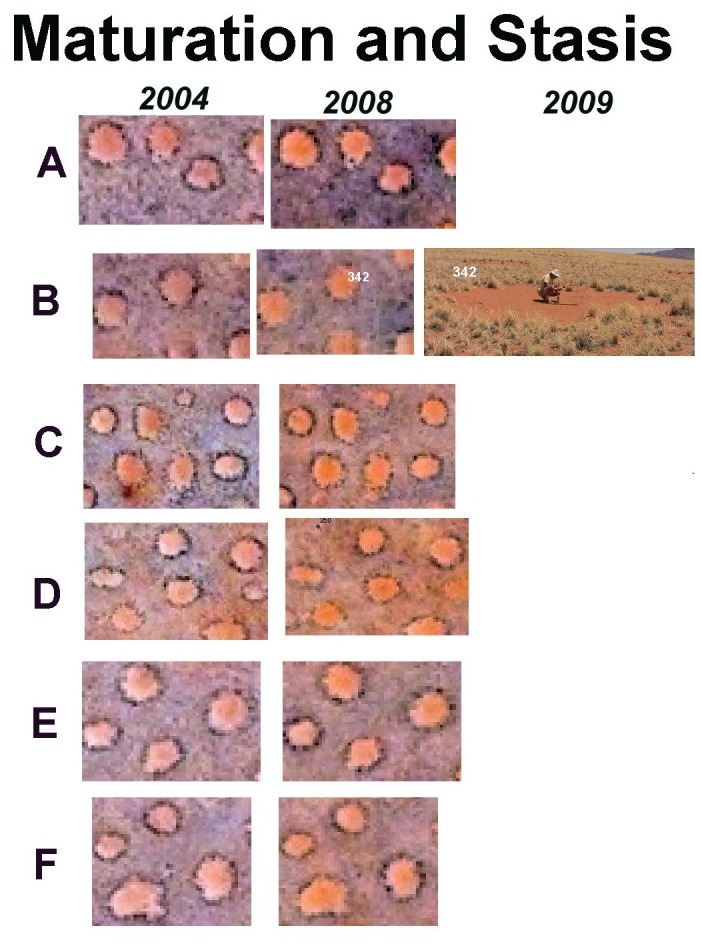
Satellite images from 2004 and 2008 showing fairy circles that did not change, and their condition of one of them upon ground checking in 2009. No specific images of other circles were made. The left and right satellite images within each row are the same scale, but the scale varies by row.

**Figure 6:**
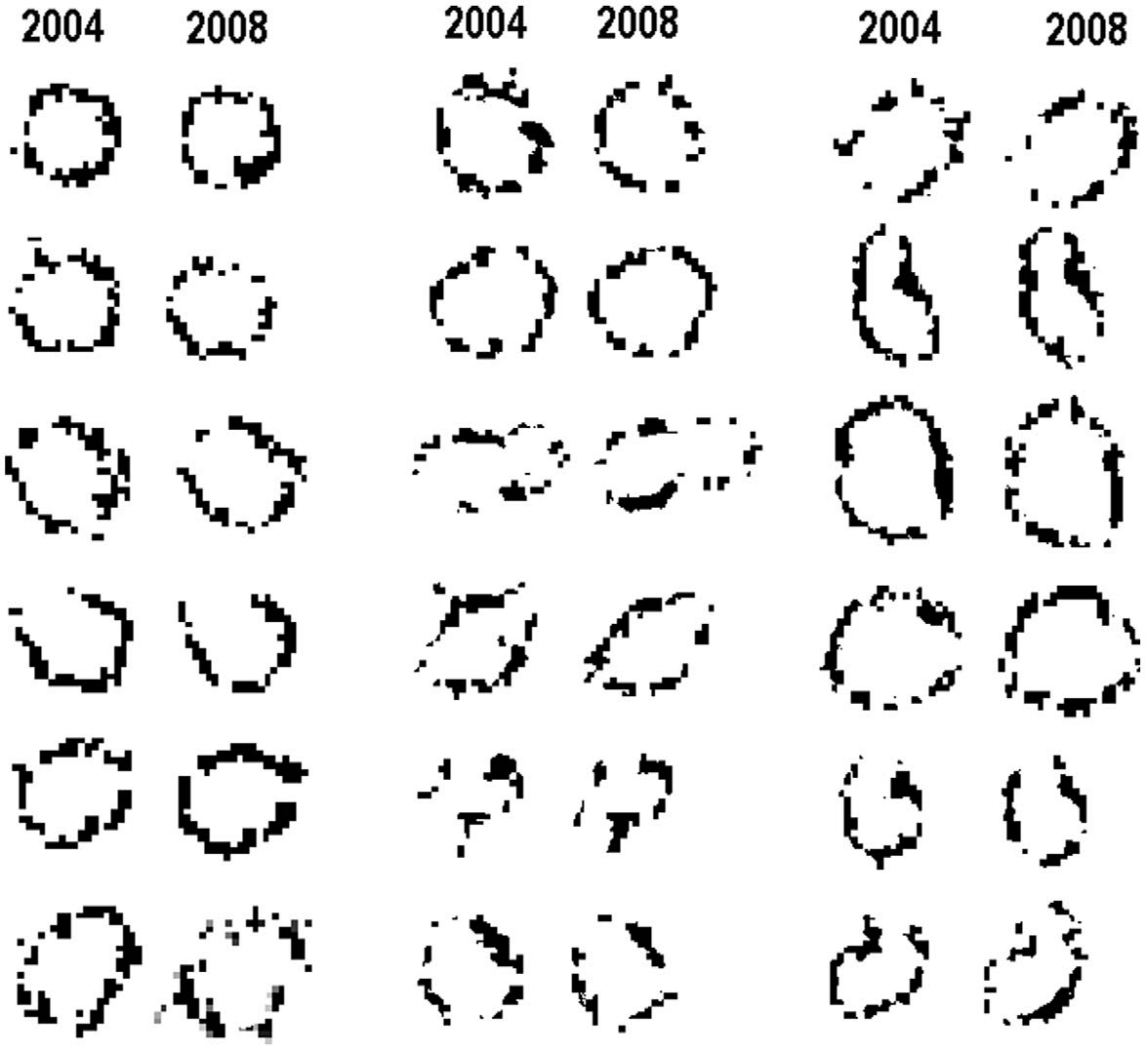
Comparison of fairy circle perimeters at high contrast in 2004 and 2008 satellite images. Circles of easily recognizable outline were chosen to show that they change little or not at all. The dark pixels are the tall grass around the circle margins.

New circles appear more or less in their final size, that is, they do not appear initially small and then grow to a mature size, or if they do, the do so in less than 4 years, the time between the two satellite photos. New circles averaged 34 m^2^ (s.d., 15; s.e. 2.3), whereas mature circles (those unchanged between 2004 and 2008) averaged 38 m^2^ (s.d. 15; s.e. 0.72) (t-test: t_477_ = 2.98; n.s.). The closeness of these two values suggests little size change over the four years. Once bare, a perimeter of tall bushman grass grows to adorn the circle, as can be clearly seen in Fig. 5. Judging from the great predominance of circles in this mature condition, they remain like this for many years, suggesting a long life span. Few such circles were ground-truthed, but Fig. 4 shows some examples of mature circles. The bareness of mature circles allows the wind to excavate them into a shallow bowl, with concavities averaging 13 cm (s.d. 5.76; maximum 23 cm) below the edges of the circle. The circles seem to change very little over long periods–even some fine details of the perimeter grass and shading are the same in 2004 and 2008 as is easily seen in Fig. 6 in which circles with distinct perimeter features are shown at extreme contrast.

**Figure 7:**
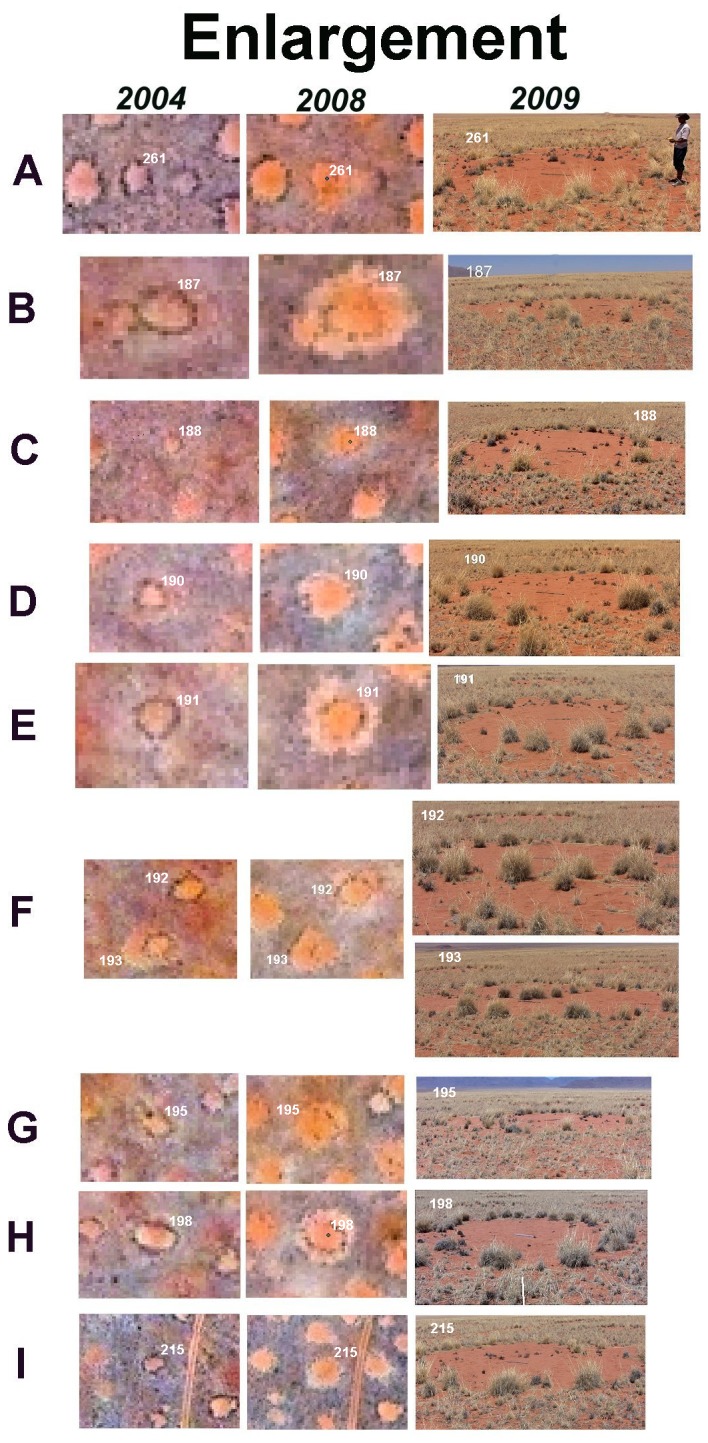
Satellite images from 2004 and 2008 showing fairy circles that grew in size, and the condition of one of them upon ground checking in 2009. The satellite images within each row are the same scale, but the scale varies by row. Each focal circle is identified by number in the photographs on the right and in the satellite images.

Occasionally, a mature circle in the 2004 image showed an enlarged reddish area beyond the dark perimeter in 2008 (Fig. 7). The enlargement was sometimes only on one side (Fig. 7A, C, F). Ground truthing such images (Fig. 7) revealed that this was indeed an enlargement in which the original perimeter grass circle now resided inside the newly bare area. In some cases, this grass was still alive, but various stages of dying were also seen. In many cases, a new, larger circle of perimeter grass had formed by the time the circle was visited in 2009 (e.g. Fig. 7A–D). Circle 193 (Fig. 7F) appears to have begun enlarging in 2004, and had formed a new ring and lost much of its original ring by 2008.

**Figure 8:**
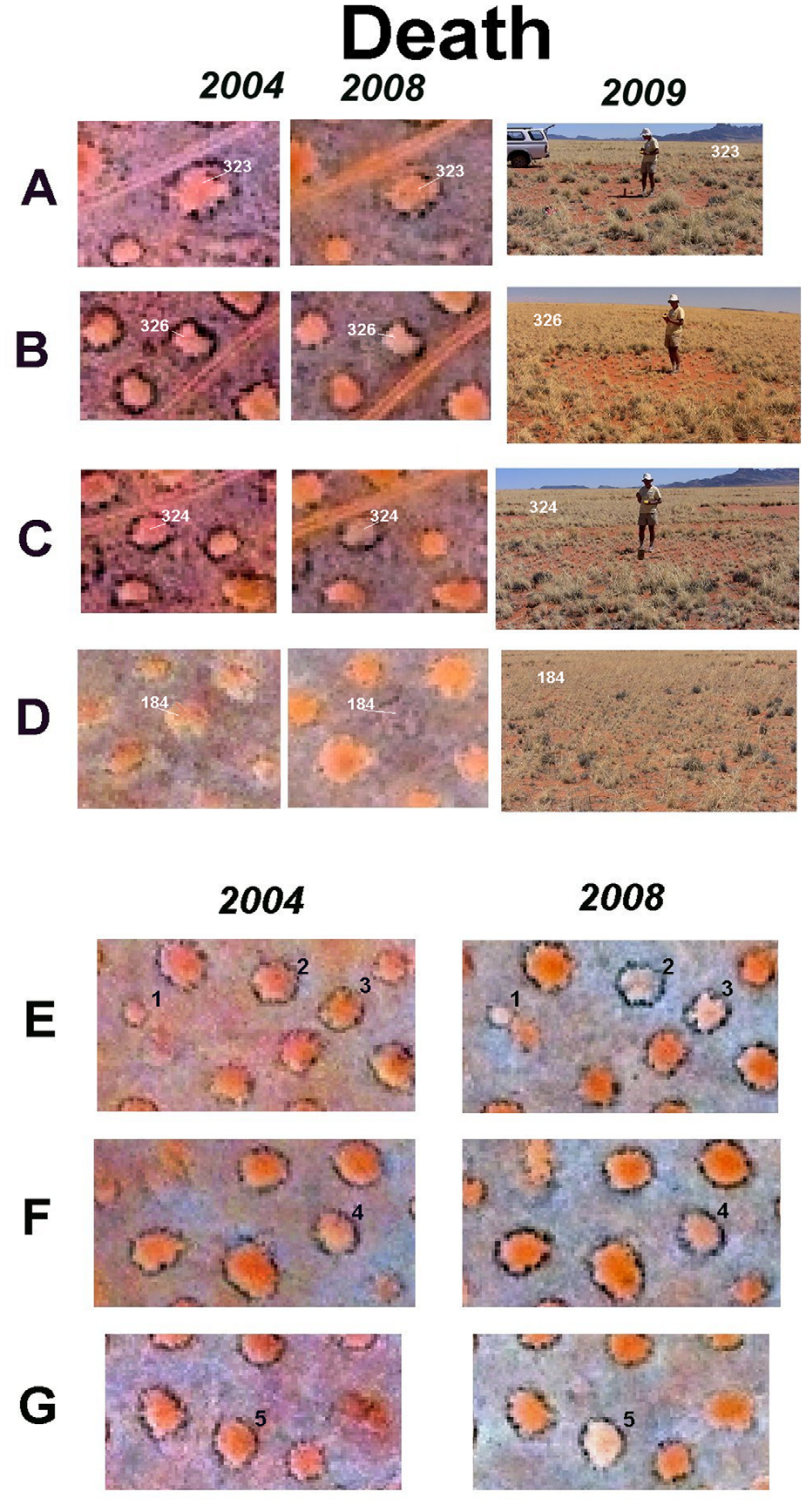
Satellite images from 2004 and 2008 showing fairy circles that were revegetated (died), and their condition upon ground checking in 2009. Additional satellite image circles are also shown without ground checked images. The satellite images within each row are the same scale, but the scale varies by row. Each focal circle is identified by number in the photographs on the right and in the satellite images.

The strongest indication of imminent death, that is, the revegetation of the bare center, was a bright, reddish center in 2004, and a distinct dulling or bluing in 2008 (Fig. 8). Because color balance varied throughout and between the images, the most telling comparison was to neighboring circles. In many cases, the ocular judgment could be confirmed by a blue-shift in red/blue ratio. Visiting circles with such a blue shift, relative to their neighbors, almost always showed them to be revegetating. Sometimes the bluing was seen in only a fraction of the pixels in the circle’s image indicating revegetation from one side.

**Figure 9:**
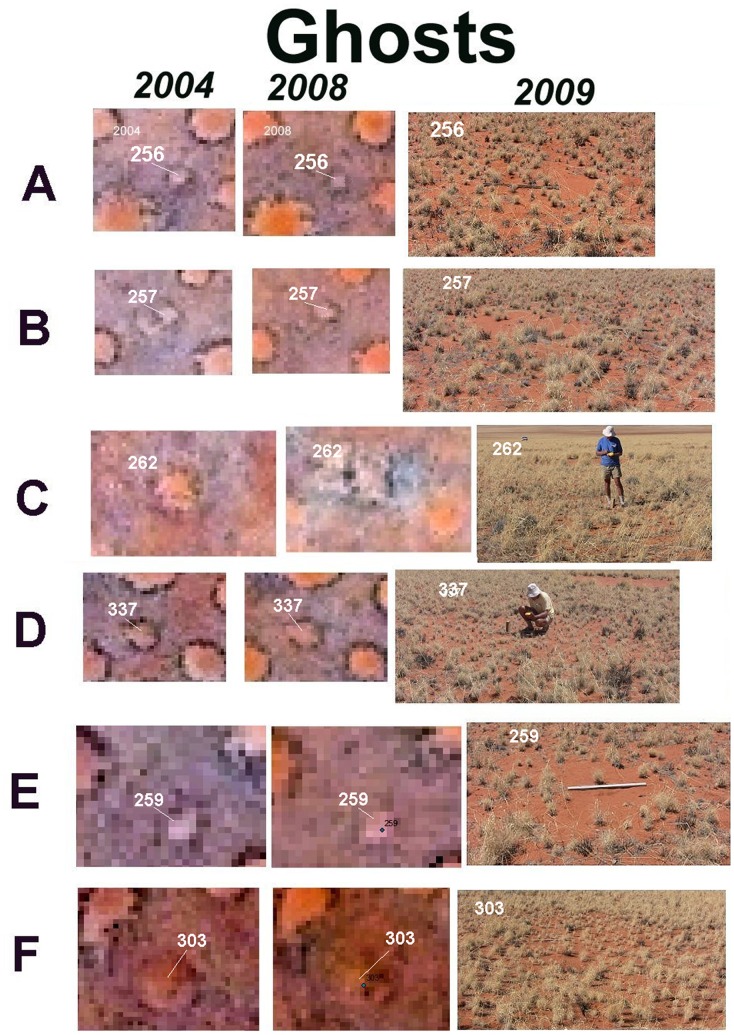
Examples of ghosts, that is, circles that appeared revegetated in both years, but were detected by the persistent ring of tall grass. Condition is shown in 2004, 2008 and upon ground checking in 2009. The satellite images within each row are the same scale, but the scale varies by row. Each focal circle is identified by number in the photographs on the right and in the satellite images.

The concavity created by the ceaseless Namibian Desert wind, along with the ring of tall grass allowed the recognition of former circles (ghosts) even after full revegetation (Fig. 9). On the ground, even after the tall grass circle had died, the concavity bore witness to the former presence of a fairy circle. The concavity is difficult to see in the photographs in Fig. 9, but it was readily spotted and measured on the ground.

Occasionally, a dying circle was immediately adjacent to a newly formed circle, usually with tangential contact. Whether there was some causal connection or just the vagaries of chance is not clear. Very rarely, a circle appeared as a ghost in 2004, but as a bright new circle in 2008, a sort of Lazarus rising-from-the-dead.

### Life History Sequence

Circle concavity vs. flatness gives a direction to the sequence described above. New circles average about 1.3 cm in concavity (s.d. 1.3; n = 14), i.e. are almost flat, mature circles average about 13 cm (s.d. 5.76; n = 20) and ghosts average 15 cm (s.d. 4.93; n = 43; maximum, 23 cm). Mature circles and ghosts are significantly more concave than new circles (ANOVA: F_2,61_ = 41.5; p<0.000001). The concavity can be created only while the circle is bare, whereas the general grass matrix is more of less flat (although undulating). Circles do not start concave and vegetated, and then proceed to flatness, rather, they start flat, lose their grass cover and proceed to concavity. It could hardly be otherwise.

### Direct Observation of Circle Formation

**Figure 10:**
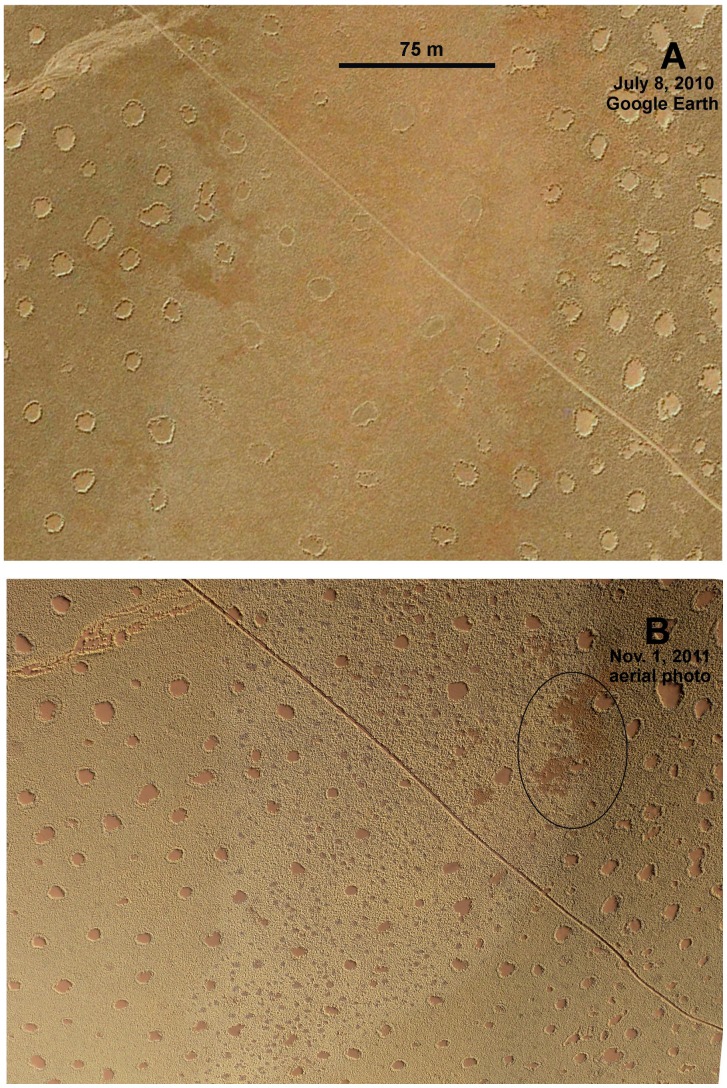
New circle formation. A. Google Earth image of July 8, 2010; B. Same area in a low altitude aerial view taken November 2011, showing new fairy circles (central region) forming in a grass matrix. Photograph courtesy of Denis Hesemans of Namib Sky Balloon Safaris.

**Figure 11:**
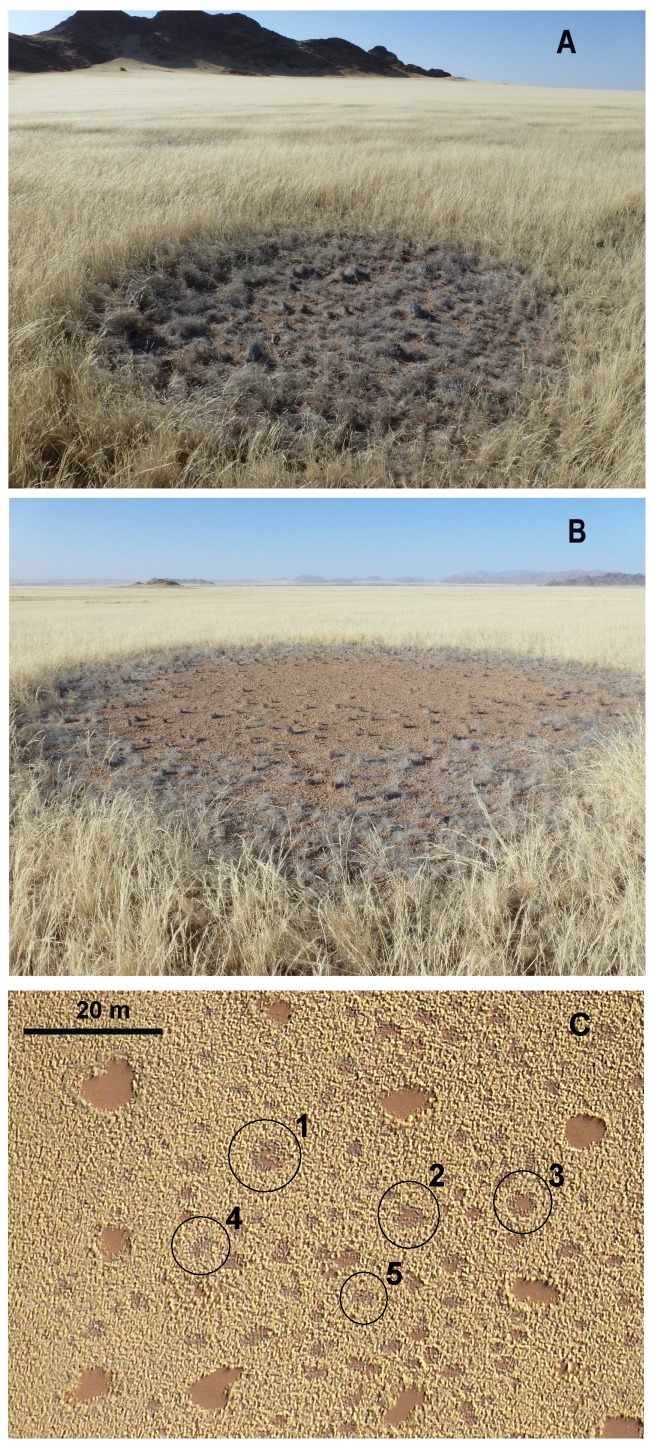
Newly forming fairy circles in a grass matrix. A. Note circle of grey, dying and collapsed grass in the upper panel, and B. its substantial disappearance in the lower panel. C. Excerpt from Fig. 10B showing aerial view of newly forming fairy circles. Photographs courtesy of Denis Hesemans of Namib Sky Balloon Safaris.

In November 2011, Denis Hesemans of Namib Sky Balloon Safaris kindly provided aerial and ground photographs of newly forming fairy circles (Figs. 10, 11) near Geluk, Namibia, about 30 km NW of the Namib Rand Nature Reserve. In Figs. 10A and B the aerial view of November 1, 2011 is compared to a Google Earth image of the same area taken July 8, 2010, one growing season earlier. In 2010, the central area was relatively uniform, with a few widely-spaced fairy circles (Fig. 10A), but by November 2011, the yellow grass of the matrix had become punctuated by grey, circular to irregular patches of dying grass, some with bare centers (Fig. 10B). The appearance of two of these patches is shown in Figs. 11A, B. In Fig. 11A, the circle is small and still covered with dead grass, whereas in Fig. 11B, dead grass in the center of the (larger) circle has disappeared, presumably through breakage and transport by wind, and the center is bare. Fig. 11C shows how such circles appear in the aerial view, with circles 1–3 showing some degree of central bareness, and 4 and 5 being still covered with dead grass. Both Figs. 11A and B are clearly new and developing fairy circles, but reconciling these images with the many grey spots in Fig. 10 is problematical. Details extracted from Fig. 10 do indeed show some spots that qualify as new fairy circles – circular patches, with dead grass and a bare center (patches 1–3 in Fig. 11C)–but these are smaller than surrounding fairy circles present in 2010 (Fig. 10A). Existing fairy circles averaged 41 m^2^ (s.d. = 27) in area, while the spots of dying grass averaged only 14 m^2^ (s.d. = 5.5). Although the minimum area was similar for both old and new circles (about 7 m^2^), the maximum size of existing circles was 173 m^2^ and that of new ones only 28 m^2^.

**Figure 12:**
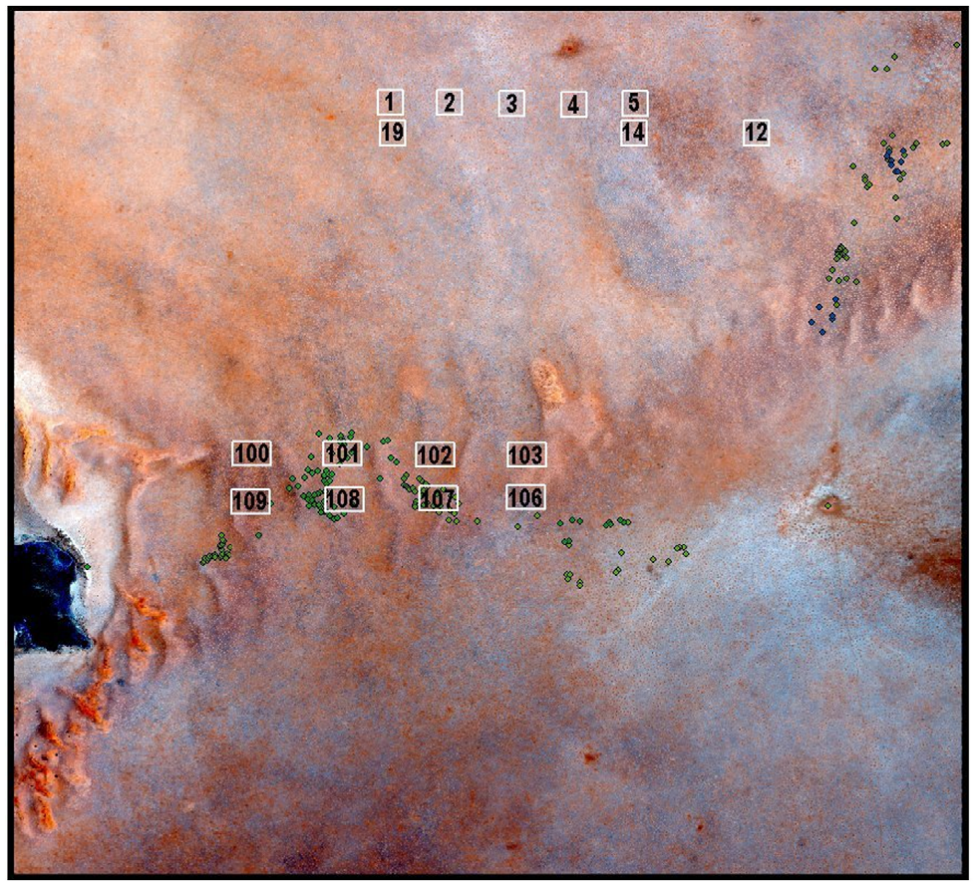
Detailed selections from the Google Earth image of the Namib Rand Nature Reserve region. Panels A-O show the variation of circle morphology, density and size. See text for details.

It seems more likely that the process seen in Fig. 10B is more than simple fairy circle formation, and that the eventual fate of this “new circle” area is not to be peppered with many tiny fairy circles, but to develop a few fairy circles within a patchy, rather disorganized matrix such as in Fig. 12A (see below) and in the ellipse in the upper right area Fig. 10B. The two areas share the characteristic of both being in the path of water flowing from surrounding hills. One could hypothesize that the small fairy circles forming in Fig. 10B would continue to grow, but this seems to be contradicted by the evidence from satellite images (above) in which fairy circles appear quickly in their final size, and rarely grow. Of course, it is possible that they grow over several years in some areas, but appear in their final size in others. Only ground checking over several years can resolve these issues.

### Estimates of the Life Span

The ground-truthed images provided the first estimate of fairy circle life span. Circles were classified into one of the following categories according to the criteria above: (1) births (absent in 2004, present in 2008); (2) stasis or enlargement (present in both years, unchanged or enlarged; (3) deaths (mature in 2004, dying in 2008). Classification was aided by ground-truthing circles in the same image, as in Figs. 5, 6, 7 and 8. Assuming that the population of circles was roughly stable, at least for the whole site, the reciprocal of the turnover rate was an estimate of the life span. Births and deaths were usually not equal, so half their sum was used as the turnover rate. However, these image areas were chosen because they contained “features of interest”, and this probably had the effect of overestimating the number of new and dying circles, i.e. the turnover. Indeed, averaged over all ground-truthed images, the estimated mean life span of about 28.6 yr (95% CI, 26.3 to 33.3 yr). Other observations (see below) suggest that this estimate is too low. Moreover, the size of these image excerpts and therefore the number of circles in them was highly variable.

**Figure 13:**
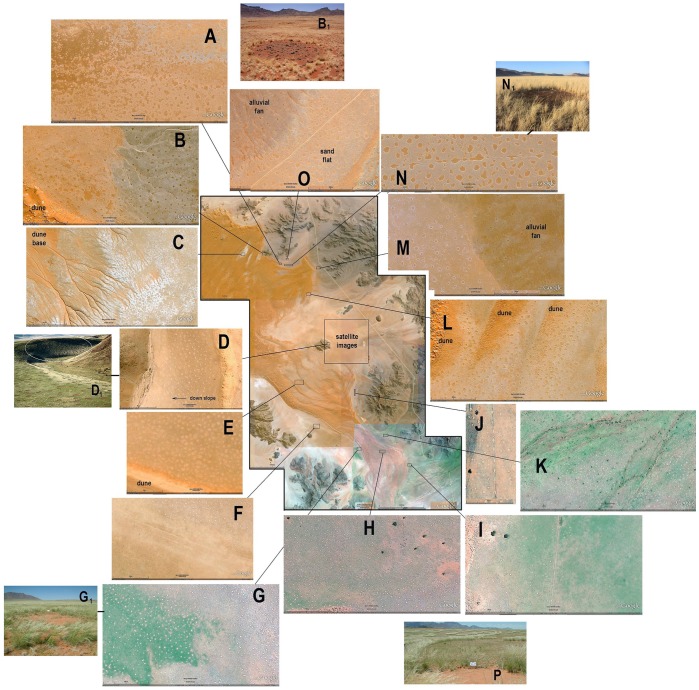
Location of the samples taken from the 2004 and 2008 images used for detailed comparison of the two years. Differences between them allowed the calculation of turnover rates and circle lifespan (see text).

A less biased test was based on 16 sites chosen from a grid, each site encompassing 176 by 90 m (15,800 m^2^) (Fig. 13, which also shows all ground-truthed features). The single condition for acceptance was that the image characteristics allowed both years to be interpreted, and the experience and judgment derived from ground-truthing could be applied. Only data with the highest level of confidence were used (605 circles). The frequencies of births, stasis and deaths are shown in Table 1. The distribution of the number of births among these grid sites did not differ from random expectation (Chi-square = 20.91; df = 15; n.s.). Deaths exceeded expectation in one image and was lower than expectation (zero) in two, resulting in a Chi-Square = 26.83; df = 15; p<0.03. However, taking births and deaths together, evidence for general regional differences in lifespan was thus weak to absent.

The means of births, deaths and stasis were used to compute a mean lifespan of 41 years, and a 95% confidence that the true mean lay between 35 and 55 years. When the data from high and medium confidence levels were used, the life spans decreased somewhat to a mean of 34 years, with 95% confidence that the true mean lay between 31 and 42 years.

Measurement of circle areas (confidence level 1 only) showed that dying circles and ghosts were smaller than new or stable circles (25 m^2^ vs. 34 and 38 m^2^, respectively; one-way ANOVA: F_2,542_ = 23.85; p<0.000001). Because circles are born at about their full size and rarely change in area once formed (Fig. 6), this suggested an alternate interpretation, namely, that smaller circles have a shorter life span. Circles were sorted into those less than 30 m^2^ (n = 195) those between 30 and 60 m^2^ (n = 318) and those greater than 60 m^2^ (n = 32) and the mean lifespan (based on turnover rate) computed for each size class. Circles of less than 30 m^2^ had a mean lifespan of about 23 yr, those 30 to 60 m^2^ lived about 75 yr and those greater than 60 m^2^ about 43 years (but sample size was small for this group). Seen this way, larger size reduced circle mortality.

### Estimation of Fairy Circle Life Span from “Sold” Circles

A total of 131 of the “sold” circles were relocated, and form the basis of a maximum likelihood estimate of lifespan. Because all circles were completely bare of vegetation when sold, any plants growing within the circles would indicate revegetation.

The maximum likelihood estimate of the fairy circle life span, β is the value β which maximizes L(β) in Equation 1. Numerically maximizing L(β) leads to an estimate of β = 59 yr. Using Equation 2, the approximate 90% and 95% confidence intervals (found by following this procedure with α = 0.10 and α = 0.05, respectively) are given by (38; 99) and (35; 111). These values are not significantly different from the estimates above. Using Equation 3, the MLE of the fraction dying annually (φ) is 0.0167, or about 1.67%, in keeping with the life span of 59 yr. Using Equation 4, the 90% and 95% confidence intervals for φ are (1.00%; 2.59%) and (0.90%; 2.79%) dying per annum.

As with the dead and dying circles in the previous analysis, those in the “sold circle” study also appeared to be smaller than those that survived. Two revegetated circles were much larger than the rest, and as a result a parametric proportional hazards analysis (Cox-type) model with area as a covariate did not detect an effect of area on circle life span, in contrast to the analysis above. This may be in part the result of the much larger sample size in the analysis based on satellite images.

### Variation in Size, Form and Density

Average circle size varied from area to area. In the thirteen 175 ×90 m^2^ samples selected from the satellite images for the analysis above (Fig. 12), individual circle size ranged from about 15 m^2^ to 133 m^2^ (4–13 m diam.) and averaged 35 m^2^ (s.d. 8.7). The mean circle size across samples ranged from 26 m^2^ to 60 m^2^, but this extreme was represented by a single sample, all others being between 26 and 40 m^2^ (approx. 6–7 m diam.). Mean circle area differed significantly among some of the samples: the single sample with a mean circle size of 60 m^2^ differed significantly from all others. A few samples with smaller circles differed significantly from others but not from each other. The number of circles in these 15,800 m^2^ samples ranged from 44 to 76, which, when combined with their areas resulted in 9 to 18% of the area being bare (mean bare area = 13% (s.d. 2.2%). Larger average circle area correlated with a greater percent bare area (R^2^ = 52%).

The analysis was extended by using Google Earth images of the wider Namib Rand Nature Reserve region to search for interesting features and patterns, and to estimate variation in form and density of fairy circles across a wider area and different geomorphology. Extracts of these images are shown at smaller scales in Fig. 12A–O. A line and rectangle shows the location of each extract in the overall image of the Nature Reserve. The scale provided on the Google Earth images was used to estimate the mean circle area, and a count of circles allowed the calculation of circle density (per hectare) and percent bare area. However, for Figs. 12A, C, I and K, these calculations did not seem useful.

Fig. 12A shows that under some conditions, neat, round, evenly-spaced fairy circles transition into a matrix of irregular, disorganized bare areas. This location is about 1 km northwest of the area in Fig. 12N. The gradual transition from organized to disorganized occurs in approximately the last half km of this distance and suggests a shift in a single process. Apparently, under some soil or other conditions, the process that normally produces circles can produce entirely different patterns.

Figs. 12B, F, M and O show contacts between primarily water-transported, alluvial material and primarily wind-transported sandy deposits. In all cases, circle size and density are larger in the sandy materials than in the alluvial, gravely material. Indeed, circles are essentially absent in the alluvial materials in Fig. 12F, as they were in many such contacts.

In Fig. 12C, runoff from dune slopes into an undrained interdune valley disrupted the regularity of fairy circle distribution and caused it to be somewhat disorganized. The braided stream beds in Fig. 11K were also devoid of circles while the area between them sustained them. In view of the location of K at the mouth of a large drainage from mountains, it seems likely that the stream bed soils were rather gravelly, while the reddish color of the areas sustaining circles suggests a veneer of windblown sand.

Fig. 12D shows that normal fairy circles can form on rather steep slopes. In this area, sand blown over the dune crest into the stream valley supports a fairly dense population of fairy circles (Fig. 12D_1_).

Figs. 12E and L demonstrate that whereas fairy circles form dense populations in the inter-dune flats, they do not populate the dunes themselves. In part this may be because the species of grasses associated with the circles do not grow on the shifting sand of dunes.

The photographs in Figs. 12G, H, I and K were taken during the summer in February 2011 after ample rains had stimulated luxurious grass growth. Note that growth is very unevenly distributed, possibly because of uneven rains or pooling of run-off. G and H were distinguished by very small fairy circles that occur at a high density, resulting in a substantial percentage of bare area (I and K were not measured). In contrast to most of the dune areas in Fig. 12, G and H support a thin forest of camelthorn acacias (*Acacia biloba*), suggesting more access to water than most other areas.

**Figure 14:**
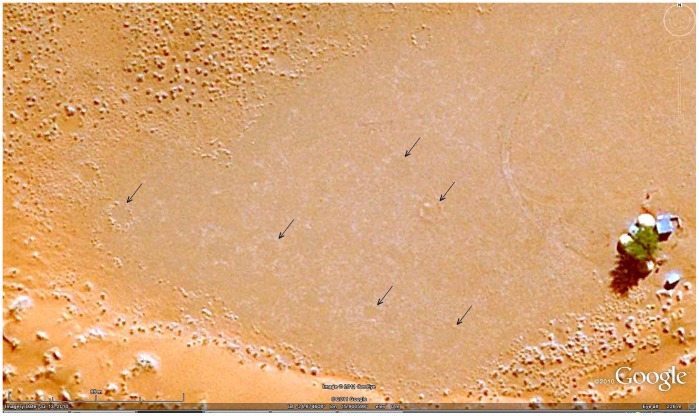
Circles sometimes lack the rim of tall grass and are formed in very sparse grass matrix, making them difficult to see in satellite images. The circles indicated by the arrows were ground checked.

Most of the fairy circles used in the previous analyses were formed in a matrix of small bushman grass with a perimeter of tall bushman grass. However, this was not universal. Some areas consisted of a continuous matrix of tall bushman grass. Circles in these areas showed no distinction between the matrix and perimeter, all being composed of tall bushman grass (Fig. 12N, N_1_). Other areas consisted of a very thin matrix of small bushman grass and lacked a perimeter of tall bushman grass. Such circles were only dimly detectable on Google Earth or satellite images (Fig. 14).

Human activities such as vehicle tracks or fences seemed able to both form and change the shape of fairy circles. Fig. 12N shows that the shapes of fairy circles was greatly elongated along an old fence line (removed decades ago), and Fig. 12J shows circles apparently formed within an abandoned vehicle track. As fences were probably also associated with vehicle tracks, perhaps some kinds of soil disturbance stimulate circle formation. It also seems possible that the elimination of vegetation competing for water allows the growth of tall bushman grass, a phenomenon visible along vehicle track in many areas. In Fig. 12P, the tall grass lining the vehicle track is clearly visible. However, many tracks and fence lines are not associated with fairy circles or tall grass margins.

Generally then, fairy circles formed primarily in sand plains, be these level, undulating or containing some stones. They were absent from shifting dunes, gravel plains and rocky areas, confirming that a “settled sandiness” is a prerequisite to the formation of fairy circles. Areas in which sands transition into mixed gravelly or other unsuitable soils often showed a decreased density, but not complete absence, of circles (Fig. 12). This suggested that the fraction of sand in soils plays a role in the process of forming fairy circles. As pure sand gives way to mixed soils, circle density decreases, eventually leading to the absence of circles. Dune sands also do not support circle formation, perhaps because the two grass species do not generally grow on shifting dunes. Occasionally, human activities, such as fence lines and roads may be associated with circles.

**Figure 15:**
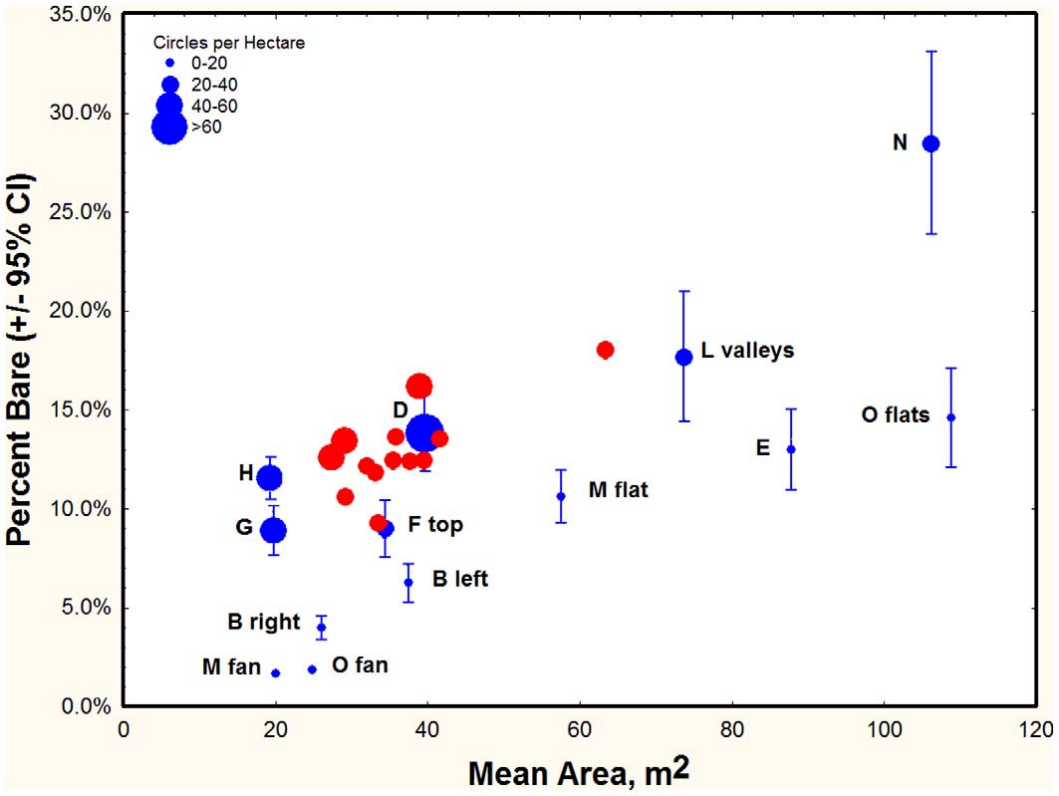
The percent of a site that was bare soil within circles increased with circle density as well as with mean circle area, but density and area changed independently of each other. Blue symbols were derived from the Google Earth selections in Fig. 11 and are labeled with the same letters as in Fig. 11. Bars are 95% confidence intervals. The red symbols are values for the selections from the 2008 satellite image used for calculating life span. Because of the large number of circles in these samples, confidence intervals were too small to show. Symbol size of both colors indicates the density of circles (see legend at upper left).

Across the selected samples for which circle size and density were determined, the percentage of the area composed of bare circles varied widely (Fig. 15, blue symbols). Both the mean circle area and the circle density (ha^−1^) simultaneously contributed to the percent bare. Every square meter increase in mean circle area was associated with 0.1% increase in percent bare, and every additional circle/ha increased this by 0.2% (multiple regression: Mean area, t_10_ = 7.45; p<0.00005; circle density, t10 = 4.37; p<0.002). It appears that circle area and density can change independently–- circle density and mean area were not significantly related, and particular mean area values were represented in a range of densities (Fig. 15). At the lowest density (6–20 ha^−1^), bareness increased almost linearly with mean area. The apparent independence of area and density suggests that separate processes are acting on these measures.

In the region of the satellite images (center box Fig. 13, Fig. 1), the density of fairy circles was much less variable, ranging from 28 to 48 ha^−1^, with a mean of 38 (S.D. 6.3) (Fig. 15, red symbols). Combining density with mean circle area, an average of 12.9% (S.D. 2.23) of the area consisted of the bare areas of fairy circles. As with density, percent bare was also much less variable than across the selections in Fig. 12. The region covered by the satellite images was a fairly uniform undulating sandy plain with a modest eastward slope running from Jagkop at the western margin to the dry lake just off the eastern margin of Fig. 2. It seems likely that the relative uniformity of this region lead to the reduced variation in fairy circle characteristics. In spite of this lower variation, the mean circle area and density were strongly related to the percent bare (multiple regression: mean area, t_10_ = 17.7; p<<0.000001; density, t_10_ = 12.1; p<<0.00001), with both factors operating simultaneously.

## Discussion

It is not the aim of this paper to explain the causes of fairy circles, but to inventory the characteristics that need explaining. Circle presence, size and spacing varied a great deal locally according to the substrate and particular area in which the circles formed. The circles are primarily a phenomenon of sandy soils, even when steeply sloped or containing stones, but are not found on gravelly or alluvial deposits or shifting dunes. Human soil disturbance such as fences and roads can change circle shape, and possibly initiate circle formation. The tall grass perimeter is not a necessary or even unique feature of fairy circles–many vehicle tracks are lined with tall grass margins and many fairy circles lack the tall grass perimeter. Because new circles appear in the short grass matrix without the tall grass perimeter, it seems likely that the bareness of circles, as of vehicle tracks, stimulates the tall grass to grow, perhaps because of reduced competition for water. In several places, soils on one side of a contact zone supported large circles at high density, while those on the other side supported small, sparse circles or none at all.

Circle area in the sites shown in Fig. 12 ranged from an average of 20 m^2^ to 110 m^2^, or about 5 m to 12 m diameter. Combining area with density of circles (range: 8 to 60 per ha), the percent of a site that was bare ranged from about 2 to 29%, increasing with either or both mean area and density. Van Rooyen et al. [2] noted that mean circle diameter decreased from about 10 m to 2 m in a southward direction from Angola to South Africa. Clearly, there is also as much or more variation within one limited region, suggesting the operation of very local as well as geographic/climatic factors.

Reliable interpretation of changes in the two satellite images depended on the focal satellite image section being similar in image quality for the two years so that differences were not readily attributable to photographic artifacts. Most of the ambiguity resided in color and contrast differences. Resolution was remarkably good and consistent, as witnessed by the pixel by pixel similarity four years apart seen in Fig. 6. This same figure also showed that most fairy circles changed very little or not at all between 2004 and 2008. When change comes, it seems to come rather quickly–circles enlarge and new circles appear in 4 years or less and often acquire a tall perimeter within 5 years or less; dying circles show the color changes of revegetation in 4 years or less and may be completely revegetated in 5.

Estimation of mean circle life span made several simplifying assumptions, most importantly uniformity in time and space. The latter assumption is probably acceptable for the relatively uniform area covered by the satellite photographs and the “sold circle” areas. All of these consist of dune fields and wind-deposited sand plains and flats. Whether circle life spans are similar on more gravelly soils or alluvial soils covered with a thin veneer of sand, or in distant regions of Namibia remains to be tested. The assumption of uniformity in time also remains to be tested, and may not hold up because it seems likely that circles are formed and revegetated following summer rains, and these are notoriously variable. For example, whereas the mean rainfall in the Namib Rand Nature Reserve is 70–80 mm per annum, 2011 saw 330 mm by the end of the austral summer in March. Moreover, the uneven distribution of green in Fig. 12G, H, I and K suggests that even very locally, rainfall may be quite variable. Strictly then, my estimates of life span apply to the changes between 2004 and 2008, but nevertheless, the fact that the “sold” fairy circles gave a similar estimate over up to 9 years suggests that these estimates are also reasonable over a longer period.

The contention by van Rooyen et al. [2] that fairy circles are more or less permanent features of the landscape has now been falsified by my data, especially by the comparison of satellite images from 2004 with those taken in 2008. Circles clearly appear and disappear, a fact that was checked many times on the ground. Albrecht et al. [1] recognized the major phases of the fairy circle life cycle, but contrary to their expectations, comparison of satellite images suggested that fairy circles do not start small and grow to mature size. Rather, they seem to appear more or less at their final size, acquiring the tall perimeter of grass in less than 5 years. Because my satellite images were separated by four years, and my visit occurred in 2009, my limit of temporal resolution was 4 years. It seems likely that new fairy circles appeared at a similar rate in each of the 4 years between the images, and they appear without the tall perimeter. New circles first visible in the 2008 satellite image could have been one to four years old. Many new circles that lacked satellite image evidence of a tall perimeter in 2008 had nevertheless acquired one by my visit in 2009, suggesting that the perimeter appears soon after the circle.

The results of this satellite image analysis need to be reconciled with those from the aerial image of November 1, 2011. If the latter represents typical fairy circle formation, then circles must grow over several years, because their initial size would be much smaller than is typical for the area. However, the fate of this area of patchy grass death is not yet apparent. It may lead to a population of small fairy circles, a disorganized, partly bare matrix or a mixture of both. At the very least, these observations reveal the very earliest stage of circle formation (Fig. 11A, B) which takes place during the periods when summer rains have stimulated luxuriant grass growth.

The potential of Google Earth, satellite and aerial images for studying fairy circles has been demonstrated here. As the resolution of Google Earth and satellite images increases, checking between images and the ground condition will provide a powerful way of tracking changes in large populations of circles. Spectral analysis, which was not available to me, would seem to hold promise for detecting more subtle changes in circles and matrix.

Although it is not the purpose of this paper to delve into the causes of fairy circles, my findings cast doubt on some of the hypotheses. Naude et al. [18] recently proposed that geological hydrocarbon seeps kill the plants. However, they did not measure hydrocarbons directly, but used carbon monoxide as a proxy. The concentrations of CO seem too low, and it is hard to picture how such seeps could consist of such evenly distributed subterranean point sources. The termite hypothesis of Albrecht et al. [1] would require that the termitaria deep underground be spaced in patterns like those of the circles above them. In principle, termite colonies have the capacity to be overdispersed, but unfortunately even the existence of such termitaria, let alone their spatial distribution is unknown. Moreover, Tschinkel [10] found no association between termite nests or foraging tunnels and fairy circles.

The highest potential for a causal mechanism of fairy circles probably lies in some form of landscape scale, self-organizing process similar to those proposed by Rietkerk et al., Couteron and LeJeune, and Tlidi et al. [11]–[16] as patterning vegetation in a range of landscapes, especially arid ones. In principle, as some limiting resource for plant growth, be it water or nutrients, becomes scarce, the capacity of plants to draw those resources toward themselves from the surrounding area creates zones of stimulated plant growth and zones where plants cannot grow, that is, the resource-accumulating process creates positive and negative feedbacks. The particular patterns then depend on the relative scale and intensity of these feedbacks. Rietkerk et al. [11]–[12] modified the cellular automaton model of Thiery et al. [15] such that nearby feedbacks were positive and distant ones negative. This version of the model created patterns that ranged from vegetation spots, to bands, tiger stripes and even holes in a matrix. While Rietkerk et al. [11], [12] cited real examples of most of the patterns created by the model (which also created fairy circle-like patterns), they appeared to be unaware of fairy circles. On the other hand, Tlidi et al. [16] specifically addressed the patterns of fairy circles in their mathematical model, and Sheffer et al. [17] tested some aspects of circle formation in the laboratory.

In contrast to several other hypotheses, self-organization is expected to create mathematically definable patterns whose scale and intensity differ under different conditions, as has been shown for fairy circles. It is more difficult to see how such processes would account for the coming and going of fairy circles. It is also not possible at this time to point to a particular resource such as water or nutrients that are the subject of the feedbacks. In an arid land, water would appear to be the most obvious choice, but Albrecht et al. [1] (2001) report that 12 days after rains, circle soil contained more water than matrix soil (but this was unreplicated, as well as a single sample point in time) and suggested that the circles act as “water traps” to benefit subterranean termites. A more meaningful test would be to compare circle and matrix soil as grass growth progresses after rains. In any case, van Rooyen et al. [2] (2004) and Moll [4] found inconsistent patterns of water content and infiltration.

Soil analysis failed to reveal any significant differences in macronutrients between matrix and circle soils. Experiments using micronutrients are currently underway (unpublished data), but results are not yet available. Whatever causes the absence of vegetation in the circles seems to affect germination little, but does not support plant growth. Van Rooyen et al. [2] (2004) and Albrecht et al. [1] (2001) also interpret the lack of growth as toxicity, their results could as readily stem from the absence of a factor needed for plant growth. In any case, the relevance of their tests is in question because they used domestic annual rye grass and Bermuda grass (respectively) rather than the native grasses that actually grow in this area of the Namib. Albrecht et al.’s [1] (2001) tests were unreplicated, used tiny amounts of soil and lawn grass instead of the grasses native to the Namib Desert. More recently, Jankowitz et al. [5] (2008) presented experiments that suggested a volatile toxic factor within fairy circles, but its possible source remains unknown. The effects of circle and matrix soils on plant growth therefore need more extensive testing and replication over longer periods. Finally, the apparent “suddenness” of grass death apparent in Fig. 11 makes causation by toxicity or disease attractive once again. Future research will have to address these hypotheses with experiments.
